# Supplementary material for: Factors associated with men’s involvement in antenatal care visits in Asmara, Eritrea: Community-based survey
Source: PLoS One. 2023 Oct 19;18(10):e0287643. doi: 10.1371/journal.pone.0287643 (PMC10586641; doi:10.1371/journal.pone.0287643)
Supplement: S1 Table — (DOCX) [file pone.0287643.s001.docx]

**S1 Table. Percentage distribution of respondents by knowledge on ANC (n=605).**

| **Variables** | **Frequency** | **Percentage** |
| --- | --- | --- |
| I have heard what ANC is | 597 | 98.7 |
| I get information about ANC from health professionals | 374 | 61.8 |
| A pregnant woman should visit antenatal clinic in the first 3 months of pregnancy | 231 | 38.2 |
| A pregnant woman should visit antenatal clinic at least 4 times during the pregnancy | 161 | 26.6 |
| A pregnant woman needs to go for ANC | 597 | 98.7 |
| A pregnant woman should attend antenatal clinic even if she has no complications. | 547 | 90.4 |
| It is necessary to take iron folic acid tablet | 297 | 49.1 |
| The mother should start taking iron folic acid tablet prior to 3 months of pregnancy. | 122 | 20.2 |
| A pregnant woman needs to take more food | 537 | 88.8 |
| A pregnant woman needs to sleep between 8-10 hours during the night time | 401 | 66.3 |
| A pregnant woman needs to sleep between 1-2 hours during the day time | 415 | 68.6 |
| Smoking is harmful to the fetus | 582 | 96.2 |
| The danger signs during pregnancy are: excessive vaginal bleeding, severe headache, swollen hands or face, epigastric pain, and blurred vision. | 132 | 21.8 |
| A pregnant woman should go to a health facility in case of an emergency | 600 | 99.2 |
| The services provided in antenatal clinic are counselling and blood testing, follow up, TT injections and urine tests. | 161 | 26.6 |
| The importance of accompanying partner to antenatal clinic are: obtaining information on both maternal and fetal conditions, improving partners’ communication, moral support, and motivating the male partner to test for HIV | 271 | 44.8 |
| I should save money for emergency when my partner is pregnant | 474 | 78.3 |
| I should arrange transportation for delivery | 503 | 83.1 |
| I should prepare essential items for clean delivery | 492 | 81.3 |
| I should arrange a blood donor in case of delivery complication | 144 | 23.8 |
| Delivered last child in hospital facilities | 599 | 99 |
