# Supplementary material for: Factors associated with men’s involvement in antenatal care visits in Asmara, Eritrea: Community-based survey
Source: PLoS One. 2023 Oct 19;18(10):e0287643. doi: 10.1371/journal.pone.0287643 (PMC10586641; doi:10.1371/journal.pone.0287643)
Supplement: S2 Table — (DOCX) [file pone.0287643.s002.docx]

**S2 Table. Percentage distribution of the respondents by attitude on ANC (n=605).**

| **Male partner's attitude toward ANC** | **Level of attitude** | |
| --- | --- | --- |
|  | **Negative %** | **Positive %** |
| A male partner should accompany his pregnant partner to ANC | 4.3 | 95.7 |
| Pregnancy is only a female domain | 81.7 | 18.3 |
| Early booking is good for the pregnancy | 4.3 | 95.7 |
| She has to go for ANC booking before the third month of pregnancy | 16.4 | 83.6 |
| Vitamin supplement is good for the fetus | 2.8 | 97.2 |
| Any amount of Alcohol drinking during pregnancy will affect growth of the unborn child | 0.8 | 99.2 |
| I will go for the ANC check-up if and only if my partner is pregnant | 43.8 | 56.2 |
| ANC follow up is good to monitor mother's and fetus' health | 4.1 | 95.9 |
| I propose for my partner to deliver in the hospital if she is pregnant | 2.8 | 97.2 |
| I will do early preparation for the delivery if she’s pregnant | 6.1 | 93.9 |
| I am ready to face my partner pregnancy and delivery complication if she’s pregnant | 8.6 | 91.4 |
| The male partner has a role during pregnancy | 7.6 | 92.4 |
| There is benefit in seeking ANC for pregnant woman | 3 | 97 |
