# Supplementary material for: Factors associated with men’s involvement in antenatal care visits in Asmara, Eritrea: Community-based survey
Source: PLoS One. 2023 Oct 19;18(10):e0287643. doi: 10.1371/journal.pone.0287643 (PMC10586641; doi:10.1371/journal.pone.0287643)
Supplement: S3 Table — (DOCX) [file pone.0287643.s003.docx]

**S3 Table. Percentage distribution of male involvement in ANC (n=605).**

| **Male's involvement in ANC** | **Frequency** | **Percentage** |
| --- | --- | --- |
| The last pregnancy of my partner was planned | 462 | 76.4 |
| My partner has visited ANC | 590 | 97.5 |
| I ever accompanied my partner to ANC | 503 | 83.1 |
| My partner has visited the ANC during her last pregnancy | 536 | 88.6 |
| I have accompanied my partner to ANC at least two times during her last pregnancy | 325 | 53.7 |
| Both me and my partner decide for her to seek ANC | 424 | 70.1 |
| Both me and partner arrange for her transportation | 472 | 78.0 |
| I escort my partner to ANC | 279 | 46.1 |
| I do discuss about pregnancy and child birth with other people | 426 | 70.4 |
