# Supplementary material for: Factors associated with men’s involvement in antenatal care visits in Asmara, Eritrea: Community-based survey
Source: PLoS One. 2023 Oct 19;18(10):e0287643. doi: 10.1371/journal.pone.0287643 (PMC10586641; doi:10.1371/journal.pone.0287643)
Supplement: S1 File — (PDF) [file pone.0287643.s004.pdf]

Questionnaire

Factors associated with men's involvement in attending antenatal care visits with their pregnant partners in Asmara, Eritrea: Community based survey

Consent

Hello: My Name is __________________. We are working in the ministry of health in Asmara. We are conducting a study on knowledge, attitude and male involvement towards ANC in Asmara. The purpose of the study is for the betterment of pregnancy outcome and to have a happy family. The information collected in this study is strictly confidential and will not be shared to others. Your participation in the study is highly crucial. However, if you are not willing to participate, you are free to leave the interview. Are you willing to participate?

Yes ☐ No ☐

Section I: Background

Sub-zoba____________ ☐ Administration Area ☐ Household Number ☐☐☐

NO.	QUESTIONS	CODING CATEGORIES	SKIP
101	Are you the head of the household?	Yes .... 1<br>No... 2	
102	How old are you?	Age ☐☐	
103	What is your current marital status?	Single .... 1<br>Married .... 2<br>Cohabiting 3	
104	What is your religion?	Christian................................. 1<br>Muslim 2	
105	What is your level of education?	Illiterate .... 1<br>Read and write .... 2<br>Elementary and junior<br>(1st-8th)................................. 3<br>High school (9th-12th)........... 4<br>Higher………………………. 5	

106	What is your current occupation?	Government employee......... A<br>Private employee....................B<br>Self-employed ....C<br>Other ___________________ X	
107	How many children do you have?	Number of Children [ ][ ]	

Section II: Knowledge

NO.	QUESTIONS	CODING CATEGORIES	SKIP
201	Have you ever heard about antenatal care?	Yes .... 1<br>No... 2	
202	Do pregnant women need to go for ANC?	Yes .... 1<br>No... 2	
203	If yes, is it required to go for ANC even if there is no complication during pregnancy?	Yes .... 1<br>No... 2	
204	At what time of pregnancy do you think is accepted for a pregnant woman to seek ANC?	First three months .... 1<br>Second three months .... 2<br>Last three months .... 3<br>Don't know 4	
205	What should be the minimum number of ANC visits during a pregnancy?	Number [ ]<br>Don't know…………………..Y	
206	What services do you know that are offered in the antenatal clinic?	Counselling and blood tests .. A<br>Observation........................... B<br>TT injections..........................C<br>Urine tests .... D<br>Other .... X<br>(specify)<br>Don't know .... Y	
207	What do you think is the importance of accompanying partner for ANC?	Get information about the pregnancy or progress of the pregnancy.............................. A<br>To improve partner communication.......................B<br>To involve male to be part of the pregnancy ....C<br>To test for HIV...................... D<br>Other .... X<br>(specify)	

208	Is it necessary for pregnant to take iron folic acid tablets?	Yes .... 1<br>No... 2 →510<br>Don't know .... 3 →510	510<br>510
209	If yes, when is it necessary to take iron folic acid tablets?	Prior to 3 months of pregnancy……………………1<br>First 3 months of pregnancy . 2<br>Next 3 months of pregnancy .. 3<br>Last 3 months of pregnancy…………………..4	
210	Do pregnant women require taking more food as compared to non-pregnant state?	Yes .... 1<br>No... 2<br>Don't know 3	
211	How many hours are required for pregnant women to sleep well during night?	Number [ ]<br>Don't know .... Y	
212	How many hours are required for pregnant women to sleep well during daytime?	Number [ ]<br>Don't know .... Y	
213	Is smoking harmful for the fetus?	Yes .... 1<br>No... 2<br>Don't know 3	
214	What are the danger signs of pregnancy?	High fever .... A<br>Severe abdominal pain...........B<br>Excessive vaginal bleeding....C<br>Fits during pregnancy .... D<br>Severe headache.................... E<br>Swollen hands and/ or face .... F<br>Loss of consciousness........... G<br>Blurred vision .... H<br>Other .... X<br>(specify)<br>Don't know .... Y	
215	Where is the place to go in case of an emergency during pregnancy?	Health facility .... A<br>Traditional healer.................. B<br>Other .... X<br>(specify)<br>Don't know .... Y	
216	Is it necessary to save money for emergency expenses?	Yes .... 1<br>No... 2	

217	What is the ideal place of delivery?	Hospital.................................. 1<br>Health center........................... 2<br>Health station/clinic .... 3<br>Home..................................... 4	
218	Is it necessary to prepare the essential items for clean delivery?	Yes .... 1<br>No... 2	
219	Is it necessary to arrange transportation for delivery?	Yes .... 1<br>No... 2	
220	Is it necessary to arrange blood donor in case of delivery complications?	Yes .... 1<br>No... 2	
221	What are your major sources of information about birth preparedness and complication readiness?	Health professionals……..…..A<br>Community health agencies……………..…….B<br>TV/Radio……………………C<br>Newspaper/ Magazines…...…D<br>Family members……………....E<br>Other .... X<br>(SPECIFY)	

Section III: Attitude

**Please, tell me whether you strongly agree, agree, medium, disagree, or strongly disagree with the following statements (1=Strongly disagree, 2=Disagree, 3=Medium, 4=Agree, 5=Strongly agree)**						
NO.	QUESTIONS	CODING CATEGORIES				
301	A male partner should accompany his pregnant partner to the ANC	1	2	3	4	5
302	Pregnancy is a female domain	1	2	3	4	5
303	Early ANC booking is good for the pregnancy	1	2	3	4	5
304	She has to go for ANC booking before the third month of pregnancy	1	2	3	4	5
305	I believe that vitamin supplement is good for the fetus	1	2	3	4	5

306	I believe alcohol drinking will affect growth	1	2	3	4	5
307	I will go for the ANC check-up if and only if my wife/ partner is pregnant	1	2	3	4	5
308	ANC follow-up is good to monitor mother's and fetus' health	1	2	3	4	5
309	I propose for my wife/ partner to deliver in the hospital if she is pregnant	1	2	3	4	5
310	I will do early preparation for the delivery if she's pregnant	1	2	3	4	5
311	I am ready to face any pregnancy and delivery complication if my wife/ partner is pregnant	1	2	3	4	5
312	The male partner has a role during pregnancy	1	2	3	4	5
313	There is benefit in pregnant woman seeking ANC	1	2	3	4	5

Section IV: Practice

NO.	QUESTIONS	CODING CATEGORIES	SKIP
401	Was the pregnancy for the last child planned?	Yes .... 1<br>No... 2	
402	Has your partner ever visited ANC services?	Yes .... 1<br>No... 2<br>Don't know 3	 →309<br>→309
403	Has your partner visited the ANC clinic during the last pregnancy?	Yes .... 1<br>No... 2	
404	Have you ever accompanied your female partner to the ANC clinic during the last pregnancy?	Yes .... 1<br>No... 2	 →306
405	How many times did you accompany her for the last pregnancy?	Number of times ☐☐	

406	Who decides for her to seek ANC?	Myself .... A<br>Herself...................................B<br>Both...................................... C<br>Parents................................. D<br>Other ___________________ X<br>(Specify)	
407	Who provides or arranges for her transportation?	Myself .... A<br>Herself...................................B<br>Both...................................... C<br>Parents................................. D<br>Other ___________________ X<br>(Specify)	
408	Who escorted her to the health facility?	Myself .... A<br>Herself...................................B<br>Her mother ....C<br>Mother-in-law .... D<br>Neighbours............................ D<br>Siblings ....E<br>Relatives............................... F<br>Other ___________________ X<br>(Specify)	
409	Do you discuss with any one about pregnancy and child birth?	Yes .... 1<br>No... 2	

THANK YOU!!!!
