# Supplementary material for: Factors associated with men’s involvement in antenatal care visits in Asmara, Eritrea: Community-based survey
Source: PLoS One. 2023 Oct 19;18(10):e0287643. doi: 10.1371/journal.pone.0287643 (PMC10586641; doi:10.1371/journal.pone.0287643)
Supplement: Attachment — Submitted filename: PONE-D-22-11568_reviewer.pdf [file pone.0287643.s005.pdf]

PLOS ONE

Factors associated with men's involvement in attending antenatal care visits with their pregnant partners in Asmara, Eritrea: Community based survey

--Manuscript Draft--

**Manuscript Number:**	PONE-D-22-11568
**Article Type:**	Research Article
**Full Title:**	Factors associated with men's involvement in attending antenatal care visits with their pregnant partners in Asmara, Eritrea: Community based survey
**Short Title:**	Factors associated with men's involvement in attending antenatal care visits with their pregnant partners
**Corresponding Author:**	Ghirmay Ghebreigziabher Beraki, MSc.<br>Orotta College of Medicine and Health Sciences<br>Asmara, Maekel ERITREA
**Keywords:**	associated factors; Male involvement; Antenatal care; Asmara
**Abstract:**	Background<br><br>Antenatal care is one of the pillars of safe motherhood by using the collective support of the health professionals, the entire family, and notably the husband/partner. Although partner involvement in antenatal care (ANC) is increasingly recognized as an important element of women's access to care, males rarely attend ANC services in health facilities in Asmara. Therefore, the study's objective was to identify the associated factors of male partners' involvement in ANC visits in Asmara.<br><br>Methods<br><br>A community-based cross-sectional survey was applied using a two-stage sampling technique to select 605 eligible respondents in Asmara in 2019. Data was collected using a pre-tested structured questionnaire. A Chi-square test was used to determine the associated factors towards male involvement in ANC care. Multivariable logistic regression was employed to determine the predictors of males' involvement in ANC. A P-value less than 0.05 was considered statistically significant.<br><br>Results<br><br>The necessity for a pregnant woman to attend ANC was recognized by almost all (98.7%) of the male partners; however, 26.6% identified the minimum frequency of ANC visits. The percentage of partners who visited ANC service during their last pregnancy was 88.6%. The percentage of male partners who scored the mean or above level of knowledge, attitude, and involvement in ANC were 57.0, 57.5, and 58.7, respectively. Religion (p=0.006), level of education (p=0.027), and level of knowledge (p<0.001) were significant predictors of male involvement in ANC.<br><br>Conclusions<br><br>Male partner involvement in ANC was considerably low. Therefore, special attention is needed for those male partners below and above secondary levels of education, with Muslim religious affiliation, and with poor knowledge on ANC to promote healthy life during pregnancy and beyond.
**Order of Authors:**	Ghirmay Ghebreigziabher Beraki, MSc.
	Hagos Ahmed, MSc
	Aster Michael, BSc
	Bereket Ghide, BSc
	Bereket Tekie Meles, BSc
	Bersabeh Tekle Tesfatsion, BSc

Powered by Editorial Manager® and ProduXion Manager® from Aries Systems Corporation

	Rida Abdulwahab, BSc
**Additional Information:**	
**Question**	**Response**
**Financial Disclosure**<br><br>Enter a financial disclosure statement that describes the sources of funding for the work included in this submission. Review the submission guidelines for detailed requirements. View published research articles from *PLOS ONE* for specific examples.<br><br>This statement is required for submission and **will appear in the published article** if the submission is accepted. Please make sure it is accurate.<br><br>**Unfunded studies**<br>Enter: *The author(s) received no specific funding for this work.*<br><br>**Funded studies**<br>Enter a statement with the following details:<br>• Initials of the authors who received each award<br>• Grant numbers awarded to each author<br>• The full name of each funder<br>• URL of each funder website<br>• Did the sponsors or funders play any role in the study design, data collection and analysis, decision to publish, or preparation of the manuscript?<br>• **NO** - Include this sentence at the end of your statement: *The funders had no role in study design, data collection and analysis, decision to publish, or preparation of the manuscript.*<br>• **YES** - Specify the role(s) played.<br><br>* typeset	The author(s) received no specific funding for this work.
**Competing Interests**<br><br>Use the instructions below to enter a competing interest statement for this submission. On behalf of all authors, disclose any competing interests that could be perceived to bias this work—acknowledging all financial support and any other relevant financial or non-financial competing interests.	The authors have declared that no competing interests exist

This statement is required for submission and **will appear in the published article** if the submission is accepted. Please make sure it is accurate and that any funding sources listed in your Funding Information later in the submission form are also declared in your Financial Disclosure statement.<br><br>View published research articles from *PLOS ONE* for specific examples.<br><br>**NO authors have competing interests**<br><br>Enter: *The authors have declared that no competing interests exist.*<br><br>**Authors with competing interests**<br><br>Enter competing interest details beginning with this statement:<br><br>*I have read the journal's policy and the authors of this manuscript have the following competing interests: [insert competing interests here]*<br><br>* typeset	
**Ethics Statement**<br><br>Enter an ethics statement for this submission. This statement is required if the study involved:<br><br>• Human participants<br>• Human specimens or tissue<br>• Vertebrate animals or cephalopods<br>• Vertebrate embryos or tissues<br>• Field research<br><br>Write "N/A" if the submission does not require an ethics statement.<br><br>General guidance is provided below. Consult the submission guidelines for detailed instructions. **Make sure that all**	Ethics approval and consent to participate<br>Ethical approval was obtained from the Eritrean Ministry of Health Ethics Committee with a ref. No.: 009/03/18 and research permission was secured from the Orotta College of Medicine and Health Sciences and Asmara Administration Office. The ethical committee approved the procedure for verbal consent for the participants. Then, the purpose, nature, potential risk, and benefit of the study were explained to the participants. The participants' rights were protected by ensuring voluntary participation and supported by verbal consent. Anonymity and privacy for the respondents were also maintained throughout, and the respondents were assured that their names would not be divulged at any point of the study. Furthermore, the participants were given a full right to drop from participating or decline to any question in the study, there will be no penalty or loss of benefits to which they are otherwise entitled.

information entered here is included in the Methods section of the manuscript.<br><br>**Format for specific study types**<br><br>**Human Subject Research (involving human participants and/or tissue)**<br>• Give the name of the institutional review board or ethics committee that approved the study<br>• Include the approval number and/or a statement indicating approval of this research<br>• Indicate the form of consent obtained (written/oral) or the reason that consent was not obtained (e.g. the data were analyzed anonymously)<br><br>**Animal Research (involving vertebrate animals, embryos or tissues)**<br>• Provide the name of the Institutional Animal Care and Use Committee (IACUC) or other relevant ethics board that reviewed the study protocol, and indicate whether they approved this research or granted a formal waiver of ethical approval<br>• Include an approval number if one was obtained<br>• If the study involved *non-human primates*, add *additional details* about animal welfare and steps taken to ameliorate suffering<br>• If anesthesia, euthanasia, or any kind of animal sacrifice is part of the study, include briefly which substances and/or methods were applied<br><br>**Field Research**<br><br>Include the following details if this study involves the collection of plant, animal, or other materials from a natural setting:<br>• Field permit number<br>• Name of the institution or relevant body that granted permission	
**Data Availability**<br><br>Authors are required to make all data underlying the findings described fully available, without restriction, and from the time of publication. PLOS allows rare exceptions to address legal and ethical	Yes - all data are fully available without restriction

Powered by Editorial Manager® and ProduXion Manager® from Aries Systems Corporation

concerns. See the PLOS Data Policy and FAQ for detailed information.<br><br>A Data Availability Statement describing where the data can be found is required at submission. Your answers to this question constitute the Data Availability Statement and **will be published in the article**, if accepted.<br><br>**Important:** Stating 'data available on request from the author' is not sufficient. If your data are only available upon request, select 'No' for the first question and explain your exceptional situation in the text box.<br><br>Do the authors confirm that all data underlying the findings described in their manuscript are fully available without restriction?	
**Describe where the data may be found in full sentences. If you are copying our sample text, replace any instances of XXX with the appropriate details.**<br><br>• If the data are **held or will be held in a public repository**, include URLs, accession numbers or DOIs. If this information will only be available after acceptance, indicate this by ticking the box below. For example: *All XXX files are available from the XXX database (accession number(s) XXX, XXX.*).<br>• If the data are all contained **within the manuscript and/or Supporting Information files**, enter the following: *All relevant data are within the manuscript and its Supporting Information files.*<br>• If neither of these applies but you are able to provide **details of access elsewhere**, with or without limitations, please do so. For example:<br><br>*Data cannot be shared publicly because of [XXX]. Data are available from the XXX Institutional Data Access / Ethics Committee (contact via XXX) for researchers who meet the criteria for access to confidential data.*	N/A

The data underlying the results presented in the study are available from (include the name of the third party and contact information or URL).<br>• This text is appropriate if the data are owned by a third party and authors do not have permission to share the data.<br><br>* typeset	
Additional data availability information:	

Powered by Editorial Manager® and ProduXion Manager® from Aries Systems Corporation

Manuscript Click here to access/download;Manuscript;Manuscript.docx

Factors associated with men's involvement in attending antenatal care visits with their pregnant partners in Asmara, Eritrea: Community based survey

Ghirmay Ghebreigziabher Beraki[1*], Hagos Ahmed[2], Aster Michael[3], Bereket Ghide[3], Bereket Tekie Meles[3], Bersabeh Tekle Tesfatsion[3], Rida Abdulwahab[3]

[1]Department of Nursing, Orotta College of Medicine and Health Sciences, Asmara, Eritrea

[2]National Statistics Office, Asmara, Eritrea

[3]Ministry of Health, Eritrea

***Corresponding author:**

Ghirmay Ghebreigziabher Beraki, Department of Nursing, Orotta College of Medicine and Health Sciences, Asmara, Eritrea. Email: gberaki83@gmail.com

Email Addresses:

HA: hagahwo2005@gmail.com

AM: astermike98@gmail.com

BG: ghidebere79@gmail.com

BTM: b.tekiemeles@gmail.com

BTT: bersabeh900@gmail.com

RA: abdulwahabrida42@gmail.com

Abstract

Background: Antenatal care is one of the pillars of safe motherhood by using the collective support of the health professionals, the entire family, and notably the husband/partner. Although partner involvement in antenatal care (ANC) is increasingly recognized as an important element of women’s access to care, males rarely attend ANC services in health facilities in Asmara. Therefore, the study's objective was to identify the associated factors of male partners’ involvement in ANC visits in Asmara.

Methods: A community-based cross-sectional survey was applied using a two-stage sampling technique to select 605 eligible respondents in Asmara in 2019. Data was collected using a pre-tested structured questionnaire. A Chi-square test was used to determine the associated factors towards male involvement in ANC care. Multivariable logistic regression was employed to determine the predictors of males’ involvement in ANC. A P-value less than 0.05 was considered statistically significant.

Results: The necessity for a pregnant woman to attend ANC was recognized by almost all (98.7%) of the male partners; however, 26.6% identified the minimum frequency of ANC visits. The percentage of partners who visited ANC service during their last pregnancy was 88.6%. The percentage of male partners who scored the mean or above level of knowledge, attitude, and involvement in ANC were 57.0, 57.5, and 58.7, respectively. Religion ($p=0.006$), level of education ($p=0.027$), and level of knowledge ($p<0.001$) were significant predictors of male involvement in ANC.

Conclusions: Male partner involvement in ANC was considerably low. Therefore, special attention is needed for those male partners below and above secondary levels of education, with

Muslim religious affiliation, and with poor knowledge on ANC to promote healthy life during pregnancy and beyond.

Keywords: Associated factors, Male involvement, Antenatal care, Asmara

Introduction

Antenatal care is a maternity care service that provides regular check-ups to treat and prevent potential health problems during pregnancy while promoting healthy lifestyles that benefit both mother and child [1]. Routine antenatal care (ANC) reduces maternal mortality and morbidity, poor pregnancy, neonatal outcomes, and other ongoing health problems [2, 3]. The World Health Organization (WHO) recommends that ANC be initiated within the first trimester of gestation with optimally eight contacts throughout the pregnancy to reduce perinatal mortality and improve women's experience of care [3, 4]. However, in 2013 the estimated coverage of early antenatal care visits was 24.0% in low-income countries compared with 81.9% in high-income countries [4].

In 2017 WHO estimated that globally 295,000 women die annually due to complications of pregnancy and childbirth. Sub-Saharan Africa and Southern Asia accounted for approximately 86% of the estimated global maternal deaths, with sub-Saharan Africa alone accounting for roughly 66% [5]. In Eritrea, the estimated maternal mortality ratio is still unacceptably high (480/100,000 live births) [5], and the significant causes of death in health facilities are sepsis and obstructed labour [6]. Therefore, to reduce the high mortality rates and have good pregnancy outcomes in developing countries like Eritrea, timely and adequate ANC visits are essential for offering multiple opportunities with numerous interventions [7]. The study has also shown that

women with at least four ANC visits were 7.3 times more likely to deliver in health facilities than those without prenatal care [7].

In the recent past, there has been a focus on engaging male partners in sexual and reproductive health (SRH) [8]. At early as 1994, at the International Conference on Population Development, it was recognized that men had a crucial role to play in the advancement of SRH [9]. The conference marked a paradigm shift in empowering women to control their fertility and have access to safe childbearing by encouraging the engagement of their partners. Since then, the focus on addressing the engagement of men has become more conceptualized in the context of SRH programs [8].

In low- and middle-income countries, men's attendance in ANC services has been found to contribute considerable health benefits for women and children [8, 10]. Moreover, researchers have found that male involvement in maternal health is important to increase the uptake of prenatal care, alleviate unnecessary delay to seek obstetric care, decrease skipping testing for HIV of his partner, and reduce stress and risk-taking behaviours of couples during pregnancy and beyond [10-15]. Therefore, the involvement of men in maternal health services is a health promotion strategy to improve maternal and child health [10, 14, 16].

Studies have recommended a need for further research, which could help explain the country-specific findings for strengthening measures to capture men’s knowledge, attitude, and behaviour related to reproductive, maternal, and child health [17]. In support of this recommendation, systematic review studies also showed that the effectiveness of interventions to encourage men to increase their attitude and knowledge in ANC resulted in more positive maternal and newborn health outcomes [10]. However, especially in sub-Saharan African countries, pregnancy and childbirth have been viewed as solely a woman’s issue, despite men having tremendous control over social and economic power over their partners [18]. Additionally, low male involvement in

maternal health care services remains a problem for healthcare providers in many African countries due to poor access, stigma, and confidentiality of services that were ‘unfriendly’ to men [19].

In Eritrea, despite 40% of all pregnant women having some complications during pregnancy and childbirth, the proportion of women who attend the first visit in the first trimester was estimated to be 22.7% [6]. As far as the authors’ level of knowledge, no published sources regarding household-based surveys around male partners’ involvement in ANC visits in Eritrea targeting the improvement of maternal health are available. Therefore, the study aims to assess the associated factors of male partners’ involvement in antenatal care services in Asmara, Eritrea.

Methods

Study design and period

A community-based cross-sectional study was carried out from April 2019 to June 2019.

Study area and population

The study was conducted in Asmara, the capital city of Eritrea. It has a population size of 420,408 and 113,413 households, according to the Asmara municipality report of 2018. The city is geographically divided into four administration blocks (clusters): southeast, southwest, northeast, and northwest. These four administration blocks had 13 sub-administration areas. One administration area was selected randomly from each cluster: Akria, Tsetserat, Arbaete-Asmara and Gejeret. Males who had pregnant partners residing in Asmara city were the study's target population.

Sample size

The sample size was determined based on Macfarlane [20], and Daniel [21] theorem using the formula: $n_1 = \frac{Z^2 P(1-P)}{e^2}$ Where n= sample size, z = 95%, C.I = 1.96, P= expected prevalence or proportion and e= precision (in proportion of one; if 5%, d= 0.05).

$$n_1 = \frac{Z^2 P(1-P)}{e^2} \qquad n_1 = \frac{(1.96)^2\, 0.50\, (1-0.50)}{(0.05)^2} \qquad n_1 = 384.16$$

The sample size was adjusted for the size of the population using the following equation:

$$n_2 = \frac{n_1 N}{N + n_1} = \frac{384.16 \times 47798}{47798+384.16} = 381.09$$

Since the sampling technique was multi-stage, a design effect (1.5) was considered then the sample size was adjusted to n_3 =571.64*(n_3= deff x n_2 = 1.5 x 381.16)*. Finally, a 6% non-response rate was added for the final sample size to be n=605 males. The non-response rate was based on the results obtained by the 2010 Eritrea Population and Health Survey (2010 EPHS) conducted by the National Statistics Office (NSO) of Eritrea, where the rate was found to range from 2.5% to 9.5% for the different regions of the country [22]. The average of this range was taken for this study.

Sampling procedure and criteria for selection

A two-stage sampling technique was adopted to select the eligible respondents from the selected administration areas. In the first stage, four administration areas, one each from the four clusters of the city, were chosen randomly, and in the second stage, the sample size for each administration area was proportionally allocated (Fig.1). The sampling interval for each administration area was calculated by dividing the number of households that fulfilled the inclusion criteria for the given sample size in that administration. Finally, the study subjects were selected by systematic random

sampling using every n^{th} interval. The first partner was determined by the lottery method, and subsequent partners were included randomly based on the breakthrough the house to house visit. Males who had pregna[illegible]artners and willing to participate were eligible for the study.

Fig 1. Flow chart of study site selection and sample allocation

Data collection tool and variable measurement

The questionnaire was developed with reference to a previous similar study [23]. After compiling the questionnaire, content validity was assessed using a panel of experts from the Reproductive Health Unit of the Ministry of Health and Midwifery Department in Asmara College of Health Sciences. This was done through cross-checking. This meant that the researcher's content validity measures contained all possible items used to measure the concept. In this study, a valid measure of 0.5 was acceptable as in the Spearman correlation coefficient. Then, experts in linguistics who are mainly involved in translating had translated the questionnaires from English to Tigrigna, the nation's local language. To further check whether the translations were consistent with originality, back translation to English were carried out. Finally, midwives experienced in research evaluated the ANC components in the questionnaire.

The questionnaire was pre-tested on 10% of the sample (61 households) in Maytemenay administration area of Asmara, which was not selected for the main study before the study period. Estimation of the time required to complete a single questionnaire and simplification of the questions that were not easily understood were mainl[illegible]erformed due to the pilot study. Finally, the questionnaire was tested for reliability using Cronbach’s Alpha statistic, which was estimated to be 0.70, which can be considered acceptable.

Data collection was done through face to face interviews with interviewers who can speak and understand the language. The modified questionnaire had four main sections. The first section collects background information, including age, address, educational level, occupation, marital status, and religion.

The second section was to assess knowledge of ANC with 21 questions consisting of questions related to information about ANC (2 items), ANC visit (4 items), sleep pattern and nutritional advice (5 items), smoking during pregnancy (1 item), danger signs during pregnancy and seeking care (2 items), services provided in ANC (1 item), accompanying the ANC (1 item), birth preparedness (4 items), and place of delivery (1 item). The answer to every item was scored as follows: “correct” (score = 1), and “wrong” (score = 0). This implies that a respondent will have a total of 21 if he had answered all questions correctly and a total score of 0 if he answered none of the questions correctly. Respondents with a total score below the mean were considered to have a poor level of knowledge, and those with a total score above the mean were considered to have a good level of knowledge on ANC.

The third section assessed attitude towards ANC with 13 questions (each with possible responses of 1=strongly disagree, 2=disagree, 3=medium, 4=agree, 5=strongly agree). Questions related to attitude on ANC: attitude on male involvement in accompanying partner to ANC (5 items), time to start ANC (2 items), planning and preparation for ANC (3 items), foodstuff good for the fetus (2 items), and benefit in pregnant woman seeking ANC (1). The total attitude score of respondents was obtained by adding scores to the 13 variables. These imply a respondent will have a total score of 65 if he strongly agrees with all the statements and a total score of 13 if he strongly disagrees with all the statements. Respondents with a total score below the mean were considered to have a

negative attitude towards ANC, while the rest with a score above the mean were supposed to have a positive attitude towards ANC.

The fourth section was to assess involvement in ANC with nine questions that included planning of pregnancy (1 items), ANC visits (6 items), and accompanying to ANC (2 items), with each question scored as 0 for no involvement and 1 for involvement. The total involvement score was determined for each respondent by adding the responses to the nine questions. This implies a respondent will have a total score of seven if he had been involved in all the ANC related statements and a total score of zero if he did not include it in all the ANC practices. Finally, respondents with a total score below the mean score were considered to have a low-level of involvement, while those above the mean were supposed to have a good level of involvement.

Data entry and analysis

After the questionnaires were manually edited and verified, they were transferred from hard copy to soft copy using a computer data entry program called Census and Survey Processing version 7 (CSPro7). Then the data was exported to SPSS version 22 for analyses. The results were summarized using frequencies and percentages. Significance of association between socio-demographic characteristics of the respondents and their level of knowledge, attitude, and involvement was carried out using Chi-square test of independence. Statistical significance was maintained when P-value was <0.05. Socio-demographic, level of knowledge, and attitude factors with $p<0.25$ were further used in the multivariable logistic regression to predict the level of male involvement in ANC. This will help identify the net impact of each factor on the level of the participation of the respondents in ANC by controlling the effect of other elements in the model.

Operational definition

Male involvement refers to a male partner visiting health facilities with his partner and physically presenting during ANC check-ups to get information abo[illegible]he pregnancy on how to support his partner for a better pregnancy and newborn outcomes.

Head of household refers to any usual adult household member who leads and manages the household regardless of gender and employment status.

Results

Socio-demographic characteristics

Data collectors were able to approach 605 males who met the inclusion criteria with a 100% response rate. The sample characteristics are presented in Table 1. The age of the respondents ranged from 25-59 years, with nearly two-thirds aged 35-54 years (63.3%). The majority of the respondents were head of households (92.2%), married (93.4%), more educated (86.1%), government employees (75.6%), and followed the Christian religion (76.9%).

Table 1. Percentage distribution of the respondents by demographic characteristics of (n=605)

Variable	Number	Percentage
**Age**		
25-34	123	20.3
35-44	201	33.2
45-54	182	30.1
55-59	99	16.4
**Head of household**		
Yes	558	92.2
No	47	7.8

**Marital status**		
Single	16	2.6
Married	565	93.4
Cohabiting	24	4.0
**Religion**		
Christian	465	76.9
Muslim	140	23.1
**Level of education**		
Illiterate	3	0.5
Read and Write	11	1.8
Elementary and Junior (1-8)	70	11.6
High School	328	54.2
Higher	193	31.9
**Respondents current occupation (n=586)**		
Government employee	443	75.6
Private employee	54	9.2
Self-employed	89	15.2
**Partner occupation (n=208)**		
Government employee	117	56.3
Private employee	47	22.6
Self-employed	44	21.2

Knowledge of the respondents towards ANC

The majority (98.7%) of the respondents had heard about ANC, and 61.8% of them received the information from health professionals. Almost all (98.7%) of the respondents understood the necessity for a pregnant woman to attend ANC and were in favour. Still, only 26.6% of them correctly identified the ideal minimum number of ANC visits.

The respondents correctly identified a pregnant woman need to: take more food (88.8%), sleep for 8-10 hours during nighttime (66.3%) and may require a nap during daytime (68.6%), and that smoking can harm the unborn child (96.2%). About half (49.1%) of the respondents were aware of the need for iron and folic acid supplements intake. However, when they were asked what time

these supplements should be taken, 20.2% answered correctly. Close to three quarters (73.4%) of the respondents did not understand services rendered during ANC visits except follow-up for pregnant woman. Almost all (99.2%) of the respondents identified health facilities as places where one should go in case of emergency; however, only 21.8% of the respondents correctly identified the danger signs of pregnancy. Only 44.8% of the respondents reported at least three of the following importance in accompanying the female partner to ANC: important in obtaining information on both maternal and fetal conditions, improving partners' communication, enhancing male involvement in the pregnancy, moral support, and motivating the male partner to test for HIV.

Respondents identified the need to save money for emergencies during pregnancy (78.3%), arranging transportation for delivery (83.1%), birth preparedness (16.1%), and preparing essential items for clean delivery (18.7%). The majority (99%) of the respondents identified the ideal place to have birth as a health facility; however, only 23.8% of the respondents mentioned the need of arranging blood donors in case of delivery complications (S1 Table).

Attitude of the respondents towards ANC

The statement with the highest percentage (99.2%) of positive attitudes reported by respondents was 'any amount of alcohol drinking during pregnancy will affect the growth of the child'. The second-highest percentage (97.0%) of the positive attitude statement reported by respondents was 'there is a benefit for pregnant women seeking ANC'. The majority (95.7%) of the respondents had a positive attitude toward the statement that a male partner should accompany his pregnant partner to ANC for early booking in the first three months; however, 81.7% of the respondents regarded pregnancy as a female domain (S2 Table).

Male partner involvement in ANC

The majority (78.0%) of the respondents aided in arranging or arranged transportation of their partners to ANC. Almost three of four (76.4%) respondents reported that the last pregnancy was planned. Just about seven of ten (70.4%) of the respondents participated in the decision made for their partners to seek ANC. Almost three of ten (29.6%) of the respondents did not discuss the topic of pregnancy and childbirth with other people, and 11.4% of respondents reported that their partners had never visited the ANC during their last pregnancy (S3 Table).

Overall, respondents' knowledge, attitude, and involvement in ANC

As shown in Table 2, 57.0%, 57.5%, and 58.7% of the respondents scored more than or equal to the mean level of knowledge, attitude, and involvement in ANC, respectively.

Table 2 Summary of overall scores on knowledge, attitude, and male partners' involvement in ANC

Variable		Cut off value	Number	Percentage
**Knowledge**				
	Below the mean	<13.9	260	43
	Mean or above	≥13.9	345	57
**Attitude**				
	Below the mean	<58.5%	257	42.5
	Mean or above	≥58.5%	348	57.5
**Involvement**				
	Below the mean	<6	250	41.3
	Mean or above	≥6	355	58.7

Bivariate associates of the level of knowledge and attitude towards ANC

Results in Table 3 show the socio-demographic characteristics considered, level of knowledge about ANC varied significantly by headship status (p=0.017). Nearly six in ten of the respondents who were heads of households had a good level of knowledge of ANC compared to four in ten of those who were not heads.

Similarly, respondents' sociodemographic characteristics, only marital status was found to have a significant association with the level of attitude of the respondents toward ANC (p=0.045). Nearly six in ten (58.7%) of the married respondents had a positive attitude towards ANC, compared to only 43.5% of those who were not married.

Knowledge of ANC has a significant contribution to having a positive attitude towards ANC (p=0.001). Close to two-thirds (63.5%) of the respondents with good knowledge of ANC were found to have a positive attitude towards ANC compared to 49.6% among those with poor knowledge.

Table 3 Bivariate associates of level of knowledge and attitude towards ANC

	Level of knowledge			Level of Attitude		
**Characteristics**	**Poor n (%)**	**Good n (%)**	**P-value**	**Negative n (%)**	**Positive n (%)**	**P-value**
**Head of household**						
Yes	232 (41.6)	326 (58.4)	**0.017**	233 (41.8)	325 (58.2)	0.215
No	28 (59.6)	19 (40.4)		24 (51.1)	23 (48.9)	
**Age**						
25-34	60 (48.8)	63 (51.2)	0.42	56 (45.5)	67 (54.5)	0.062
35-44	79 (39.3)	122 (60.7)		74 (36.8)	127 (63.2)	
45-54	79 (43.4)	103 (56.6)		75 (41.2)	107 (58.8)	
55-59	42 (42.4)	57 (57.6)		52 (52.5)	47 (47.5)	
**Marital Status**						

Married	237 (42.4)	322 (57.6)	0.317	231 (41.3)	328 (58.7)	**0.045**
Others	23 (50)	23 (50)		26 (56.5)	20 (43.5)	
**Religion**						
Christian	192 (41.3)	273 (58.7)	0.127	196 (42.2)	269 (57.8)	0.766
Muslim	68 (48.6)	72 (51.4)		61 (41.6)	79 (56.4)	
**Level of education**						
Below secondary	37 (44)	47 (56)	0.888	37 (44)	47 (56)	0.677
Secondary	138 (42.1)	190 (57.9)		143 (43.6)	185 (56.4)	
Higher	85 (44)	108 (56)		77 (39.9)	116 (60.1)	
**Respondents current occupation**						
Government employee	192 (43.3)	251 (56.7)	0.764	185 (41.8)	258 (58.2)	0.554
Non-governmental employees and unemployed	68 (42)	94 (58)		72 (44.4)	90 (55.6)	
**Level of Knowledge**						
Poor				131 (50.4)	129 (49.6)	**<0.001**
Good				126 (36.5)	219 (63.5)	
**Total**	**260**	**345**		**257**	**348**	

Bivariate and multivariable predictors of male partners' involvement in ANC.

Table 4 signifies the association of male involvement with selected socio-demographic characteristics, level of knowledge, and attitude of the respondents. The bivariate analysis revealed that religion (p=0.010), level of education (p=0.001), level of knowledge (p<0.001), and level of attitude (p=0.049) of the respondents were found to be associated significantly with their involvement in ANC.

At the multivariable level, variables that had a p-value less than 0.25 were included due to their potential confounding effect. The result at the multivariable level revealed that religion (p=0.006), a secondary level of education (p=0.027), and a good level of knowledge (p<0.000) as significant predictors of male involvement in ANC.

Knowledge of ANC was found to have a significant positive contribution toward involvement in ANC. Respondents with good knowledge of ANC were 3.8 times more likely to be involved in ANC compared to those with a poor level of knowledge [AOR: 3.80, 95% CI: 2.46, 5.87]. The odds of being involved in ANC were nearly 2 times higher among those with a secondary level of education than those who attended below the secondary level of education taken as a reference category [OR: 1.96, 95% CI: 1.08 3.57]. The likelihood to participate in ANC was almost two times higher among Christian respondents than Muslims (OR: 1.91, 95% CI: 1.20, 3.03].

The multivariable analysis result further indicated that the regression model is statistically significant in predicting the likelihood of involvement of the respondents based on their level of knowledge, level of education, and religion[χ^2(5)= 66.99, p<0.001]. The model correctly predicted the likelihood of involvement of males in ANC for 79.7% of the respondents.

Table 4. Bivariate and multivariable predictors of male partners' involvement in ANC.

Variable	Bivariate Analysis			Multivariable Analysis	
	Poor (n=250)	Good (n=355)	p-value	AOR (95% CI)	p-value
**Head of Household**					
Yes	40.9	59.1	0.426	-	-
No	46.8	53.2			
**Age**					
25-34	35.8	64.2	0.069	1.56 (0.78, 3.12)	0.209
35-44	36.8	63.2		1.59 (0.83, 3.05)	0.162
45-54	47.8	52.2		0.82 (0.44, 1.51)	0.522
55 -59	45.5	54		Reference	
**Current Marital Status**					
Married	41.9	58.1	0.349		
Others	34.8	65.2		-	-
**Religion**					

Christian	38.5	61.5	**0.010**	1.91 (1.20, 3.03)	**0.006**
Muslim	50.7	49.3		Reference	
**Level of Education**					
Below secondary	57.1	42.9		Reference	
Secondary	35.7	64.3	**0.001**	1.96 (1.08, 3.57)	**0.027**
Higher	44	56		1.23 (0.66, 2.28)	0.520
**Respondents current occupation**					
Government employee	40	60			
Other employees and unemployed	45.1	54.9	0.259	-	-
**Level of Knowledge**					
Poor	58.1	41.9	**<0.001**	Reference	**<0.001**
Good	28.7	71.3		3.80 (2.46, 5.87)	
**Level of Attitude**					
Negative	45.9	54.1	**0.049**	Reference	0.289
Positive	37.9	62.1		1.26 (0.82, 1.92)	
**Overall Percentage**	41.3	58.7			

Discussion

The role of men in women's sexual and reproductive health outcomes has been increasingly recognized internationally since the mid-1990s [24]. Studies have also shown that pregnancy outcomes and child development improve when men are directly involved in maternal health care [25, 26]. Furthermore, when men are partners during ANC, they are supportive socially, economically, and emotionally, essential in improving maternal and newborn health outcomes [27]. Therefore, expanding initiatives to include male partners and promoting their involvement in ANC could be considered an important strategy to go beyond perinata[illegible]ealth [2].

This study considers the perspectives of male partner involvement in ANC and highlights the constraints and concerns faced by them. The remarkable proportion of the respondents with a low level of knowledge (43%), attitude (42.5%) and male partner involvement (41.3%) on ANC are the main concerns and constraints based on the results of our study. Hence, to address this

constraint, perinatal health education interventions are needed for male partners in all reproductive age categories, educational status below and above secondary levels, and those with Muslim religious affiliation in Asmara.

This insight can help in reorienting our ANC service to be more need-based and couple oriented than exclusively focused on women in maternal health programs that are inaccessible to men. In the Eritrea health care system, community health agents have active participation in the community to promote a healthy lifestyle in the community [28]. However, about three-fourths of the respondents of this study did not know any ANC services, and this showed that health education programs are not orienting men to share equal responsibilities during pregnancy to improve the maternal health outcome. Besides, the majority (81.7%) of the study participants also responded that ‘pregnancy is a female domain’.

In this study, the percentage of partners with a good level of knowledge was 57.0% and a similar finding was noted in a study conducted in Iran [29]. However, in our study only about a quarter (21.8%) of the respondents knew the danger signs of pregnancy in antenatal clinics as compared to that of Nepal (39.3%) [30]. In contrast to our finding, in a study conducted in Ethiopia, slightly more than half of the respondents knew the danger signs of pregnancy [23]. This variation in findings could be due to differences in the community's cultural context, level of education, and nature of ANC services rendered in the countries.

Our study demonstrates that level of knowledge has a significant relationship with male involvement in ANC, similar to the finding of the studies conducted in Myanmar [26] and Tanzania [31]. Moreover, a significant association was also observed between the respondents' level of knowledge and attitude in ANC. Therefore, it is advisable to invest in knowledge of the husbands/partners on ANC to increase their involvement in ANC.

This cross-sectional study identified that 57.5% of the respondents had a positive attitude towards ANC, which is less than the studies in Ethiopia (73.8%) [23] and Iran (65.3%) [29] but in line with that of Nigeria (56.5%) [32]. Furthermore, the findings of this study suggest that attitude had a significant association with the level of knowledge which is incomparable with the result in Nigeria, where the attitude was significantly associated with the level of education [32].

The main reason for having the average attitude level of the respondents in this study could be the influence of a culture that considers maternal health as a women’s domain, lack of services targeting men, and the lack of privacy at the clinics. Therefore, there is a need to change men’s attitudes to better understand maternal health care outcomes because men are the main decision-makers of health care in families in Eritrea.

The level of involvement in this study (58.7%) is similar to other studies in Ethiopia (59.9%) [23] and Tanzania (56.9%) [31] but higher than the one found in Nepal, which estimated men's involvement at 39.3% [30]. Our study also revealed that 53.7% of the respondents accompanied their partners to antenatal clinics two or more times, which is similar to the study conducted in Northern Uganda [31] and higher than in Nepal [30]. Moreover, in our study, 81% of women were accompanied to ANC clinics by their partners at least one time instead of 47% in Bangladesh [33] and 68% in Kenya [34]. The element contributing to this high male involvement in ANC in our study could be the introduction of couple counselling and testing for HIV during ANC. Regarding who decided to seek health care, 80.3% of the couples decided both in our study. Whereas in the study conducted in Ambo Town of Ethiopia, 60.7% of the partners decided alone [23], and in Western Australia, men had no role in decision making [35].

This study revealed that religion had a strong association with male involvement in ANC, men of the Christian faith reported relatively higher involvement in ANC than their Muslim counterparts.

Studies conducted in Tanzania [14], Nigeria [13] and Cameroon [36] also had similar findings. Therefore, it is advisable to consider religious organizations as a platform to support males to participate more in maternal health.

This study has shown that level of involvement was associated with religion and educational level; similarly, in the study conducted in Nepal, academic status and age were associated with male participation in ANC [30]. Conversely, in the study conducted in Eastern Ethiopia, male involvement was proved to have no association with educational level or occupational status but associated with the occupational status of the wife [37].

Men's involvement in ANC occurs more when men have good knowledge and positive attitudes toward the health of their partners during ANC [17]. The present study supported these findings and religion and level of education as significant predictors of male involvement in ANC. Based on our results, we recommend local policymakers and programmer developers use our study's main indicators to make male participation in maternal health more effectively than ANC's traditional interventions that only target women in Eritrea [14].

Strengths and limitations

The study's main strength is that it is the first study focusing on affecting factors in male partner involvement in ANC services in Asmara. Moreover, the study's main strength was an adequate sample size to determine the associated factors of men's participation. However, the study is cross-sectional, where there is a difficult to draw causal inferences.

Conclusions

The study showed that close to sixty percent of the respondents had a good level of knowledge, attitude, and involvement in ANC. A good level of knowledge was among the main predictors of

high participation in ANC. Moreover, most of the respondents believed that pregnancy is a female domain. Hence, this belief and the poor knowledge, can pose a challenge to the involvement of males in ANC. Therefore, there is a need to create an outlook that favours an equal share of responsibilities during pregnancy, especially focusing on male partners in all reproductive age categories, those with below and above secondary levels of education, those with Muslim religious affiliation, and those with a poor level of knowledge on ANC.

Abbreviations

ANC: Antenatal Care/Clinic; **HIV**: Human Immunodeficiency Virus; **SPSS:** Statistical Package for Social Sciences; **WHO:** World Health Organization

Declarations

Ethics approval and consent to participate

Ethical approval was obtained from the Eritrean Ministry of Health Ethics Committee with a ref. No.: 009/03/18 and research permission was secured from the Orotta College of Medicine and Health Sciences and Asmara Administration Office. The ethical committee approved the procedure for verbal consent for the participants. Then, the purpose, nature, potential risk, and benefit of the study were explained to the participants. The participants' rights were protected by ensuring voluntary participation and supported by verbal consent. Anonymity and privacy for the respondents were also maintained throughout, and the respondents were assured that their names would not be divulged at any point of the study. Furthermore, the participants were given a full right to drop from participating or decline to any question in the study, there will be no penalty or loss of benefits to which they are otherwise entitled.

Consent for publication

Not applicable

Availability of data and materials

The complete dataset used and analyzed at the present study are available in electronic and paper form with the corresponding author and can be accessed upon a reasonable request.

Competing interests

The authors declare that they have no competing interests.

Funding

No funds were available.

Author Contributions

Conceptualization: Ghirmay Ghebreigziabher Beraki, Aster Michael, Bereket Ghide, Bereket Tekie Meles.

Data collection: Aster Michael, Bereket Ghide, Bereket Tekie Meles, Bersabeh Tekle Tesfatsion, Rida Abdulwahab

Formal analysis: Ghirmay Ghebreigziabher Beraki, Hagos Ahmed.

Methodology: Hagos Ahmed

Validation: Ghirmay Ghebreigziabher Beraki, Bersabeh Tekle Tesfatsion, Rida Abdulwahab

Writing – original draft: Aster Michael, Bereket Ghide, Bereket Tekie Meles, Bersabeh Tekle Tesfatsion, Rida Abdulwahab

Writing – review & editing: Ghirmay Ghebreigziabher Beraki. Hagos Ahmed, Aster Michael, Bereket Ghide, Bereket Tekie Meles, Bersabeh Tekle Tesfatsion, Rida Abdulwahab

Acknowledgements

The authors are grateful for the voluntary participation and cooperation of the respondents. We appreciate the support of the administration staff of municipality office of Asmara for allowing us to have access to conduct house to house survey.

Supportive information

S1 Table. Percentage distribution of respondents by knowledge on ANC (n=605).

S2 Table. Percentage distribution of the respondents by attitude on ANC (n=605).

S3 Table. Percentage distribution of male involvement in ANC (n=605).

S1 File. Questionnaire (PDF)

Reference

1. Banta, D., *What is the efficacy/effectiveness of antenatal care and the financial and organizational implications?* . 2003: WHO Regional Office for Europe; Copenhagen
2. WHO, *WHO recommendation on health promotion interventions for maternal and newborn health 2015.* 2015, Geneva: World Health Organization.
3. WHO, *WHO recommendations on antenatal care contact schedules.* 2020, Geneva: World Health Organization.
4. Moller, A.-B., et al., *Early antenatal care visit: a systematic analysis of regional and global levels and trends of coverage from 1990 to 2013.* The Lancet Global Health, 2017. **5**(10): p. e977-e983.
5. WHO, *Trends in maternal mortality: 2000-2017: estimates from WHO, UNICEF, UNFPA, World Bank Group and the United Nations Population Division*. 2017, Geneva: World Health Organization.
6. MOHE, *Health Management Information System (HMIS): Annual health service report activity 2019. Health Management Information System (HMIS) report* 2020, MOHE: Asmara, Eritrea.
7. Guliani, H., A. Sepehri, and J. Serieux, *What impact does contact with the prenatal care system have on women's use of facility delivery? Evidence from low-income countries.* Social science & medicine, 2012. **74**(12): p. 1882-1890.

8. Ruane-McAteer, E., et al., *Interventions addressing men, masculinities and gender equality in sexual and reproductive health and rights: an evidence and gap map and systematic review of reviews.* BMJ global health, 2019. **4**(5): p. e001634.
9. Platiner, M., *The status of women under international human rights law and the 1995 un world conference on women, Beijing, China.* Ky. LJ, 1995. **84**: p. 1249.
10. Tokhi, M., et al., *Involving men to improve maternal and newborn health: a systematic review of the effectiveness of interventions.* PloS one, 2018. **13**(1): p. e0191620.
11. Aarnio, P., et al., *Male involvement in antenatal HIV counseling and testing: exploring men's perceptions in rural Malawi.* AIDS care, 2009. **21**(12): p. 1537-1546.
12. Kululanga, L.I., J. Sundby, and E. Chirwa, *Male involvement in maternity health care in Malawi.* African journal of reproductive health, 2012. **16**(1): p. 145-157.
13. Iliyasu, Z., et al., *Birth preparedness, complication readiness and fathers' participation in maternity care in a northern Nigerian community.* African journal of reproductive health, 2010. **14**(1).
14. Gibore, N.S., T.A. Bali, and S.M. Kibusi, *Factors influencing men's involvement in antenatal care services: a cross-sectional study in a low resource setting, Central Tanzania.* Reproductive health, 2019. **16**(1): p. 52.
15. Ongolly, F.K. and S.A. Bukachi, *Barriers to men's involvement in antenatal and postnatal care in Butula, western Kenya.* African journal of primary health care & family medicine, 2019. **11**(1): p. 1-7.
16. Byamugisha, R., et al., *Male partner antenatal attendance and HIV testing in eastern Uganda: a randomized facility-based intervention trial.* Journal of the International AIDS Society, 2011. **14**(1): p. 43.
17. Assaf, S., and Lwendo Moonzwe Davis. , *Does Men's Involvement Improve the Health Outcomes of Their Partners and Children?* 2018, DHS Analytical Studies No. 46: Rockville, Maryland, USA: ICF.
18. Chibwae, A., et al., *Attendance of male partners to different reproductive health services in Shinyanga District, North western Tanzania.* Tanzania Journal of Health Research, 2018. **20**(2).
19. Bwambale, F.M., et al., *Voluntary HIV counselling and testing among men in rural western Uganda: implications for HIV prevention.* BMC public health, 2008. **8**(1): p. 1-12.
20. Macfarlane, S.B., *Conducting a descriptive survey: 2. Choosing a sampling strategy.* Tropical doctor, 1997. **27**(1): p. 14-21.
21. Daniel, W.W., *Biostatistics: Basic Concepts And Methodology For The Health Sciences, Isv.* 2010.
22. NSO, *Eritrea Population and Health Survey 2010*, 2010, Editor. 2013, National Statistics Offce and Fafo Institute for Appled: Asmara, Eritrea: .
23. Demissie, D.B., G.A. Bulto, and T.G. Terfassa, *Involvement of male in antenatal care, birth preparedness and complication readinessand associated factors in Ambo town, Ethiopia.* J Health Med Nurs, 2016. **27**(5): p. 14-23.
24. Greene, M.E. and A.E. Biddlecom, *Absent and problematic men: Demographic accounts of male reproductive roles.* Population and development review, 2000. **26**(1): p. 81-115.
25. Redshaw, M. and J. Henderson, *Fathers' engagement in pregnancy and childbirth: evidence from a national survey.* BMC pregnancy and childbirth, 2013. **13**(1): p. 70.

26. Ampt, F., et al., *Correlates of male involvement in maternal and newborn health: a cross-sectional study of men in a peri-urban region of Myanmar.* BMC pregnancy and childbirth, 2015. **15**(1): p. 122.
27. Yargawa, J. and J. Leonardi-Bee, *Male involvement and maternal health outcomes: systematic review and meta-analysis.* J Epidemiol Community Health, 2015. **69**(6): p. 604-612.
28. EMOH, *Safe mother hood emergency obstetrics and new born care curriculum; basic lifesaving skills*. 2nd ed. 2011: Sabur printing press.
29. Farzaneh Soltani, M.M., Fatemeh Shobeiri, Parisa Parsa, Ghodratollah Roshanaei, *Knowledge and Attitude of Men towards Participation in Their Wives' Perinatal Care.* International Journal of Women's Health and Reproduction Sciences, 2018. **6**(3): p. 356–362.
30. Bhatta, D.N., *Involvement of males in antenatal care, birth preparedness, exclusive breast feeding and immunizations for children in Kathmandu, Nepal.* BMC pregnancy and childbirth, 2013. **13**(1): p. 14.
31. Kabanga, E., et al., *Prevalence of male partners involvement in antenatal care visits–in Kyela district, Mbeya.* BMC pregnancy and childbirth, 2019. **19**(1): p. 1-6.
32. Olugbenga-Bello Adenike, I., et al., *Perception, attitude and involvement of men in maternal health care in a Nigerian community.* Journal of Public Health, 2013. **5**(6): p. 262-270.
33. Rahman, A.E., et al., *Knowledge and involvement of husbands in maternal and newborn health in rural Bangladesh.* BMC pregnancy and childbirth, 2018. **18**(1): p. 1-12.
34. Kwambai, T.K., et al., *Perspectives of men on antenatal and delivery care service utilisation in rural western Kenya: a qualitative study.* BMC pregnancy and childbirth, 2013. **13**(1): p. 1-10.
35. Jeffery, T., et al., *AUSTRALIAN FATHER'S STUDY: WHAT INFLUENCES PATERNAL ENGAGEMENT WITH ANTENATAL CARE?: P025.* Journal of Paediatrics and Child Health, 2014. **50**: p. 90-91.
36. Nkuoh, G.N., et al., *Barriers to men's participation in antenatal and prevention of mother-to-child HIV transmission care in Cameroon, Africa.* Journal of midwifery & women's health, 2010. **55**(4): p. 363-369.
37. Asefa, F., A. Geleto, and Y. Dessie, *Male partners involvement in maternal ANC care: the view of women attending ANC in Hararipublic health institutions, eastern Ethiopia.* Sci J Public Health, 2014. **2**(3): p. 182-8.

Figure

Click here to access/download;Figure;Fig. 1.docx

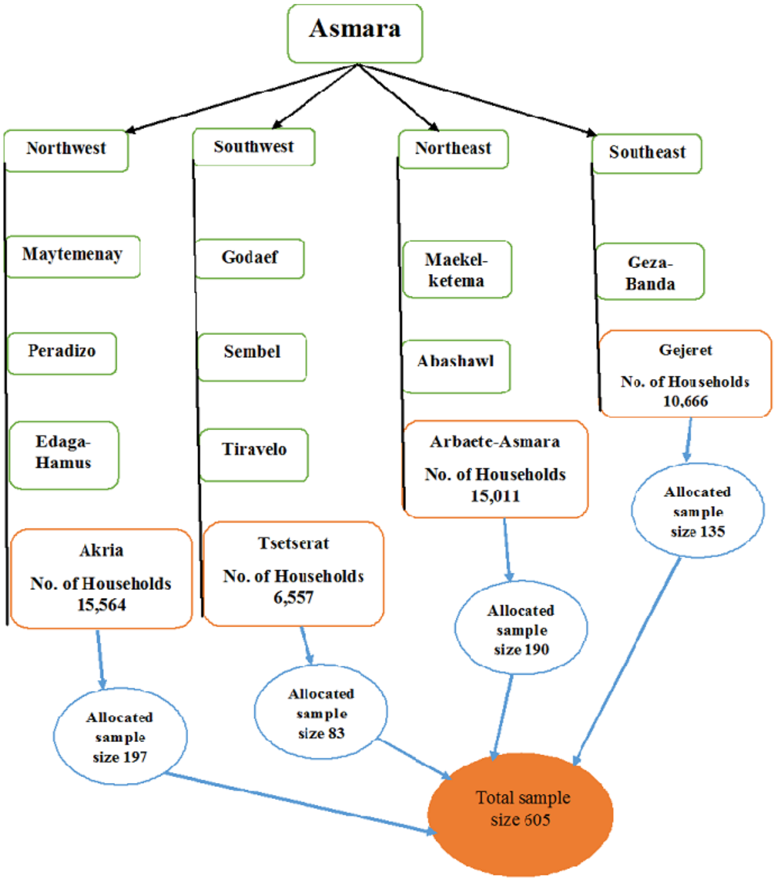

Click here to access/download
Supporting Information
Supplementary Tables.docx

Click here to access/download
Supporting Information
Questionnaire.pdf
