# Supplementary material for: Factors associated with men’s involvement in antenatal care visits in Asmara, Eritrea: Community-based survey
Source: PLoS One. 2023 Oct 19;18(10):e0287643. doi: 10.1371/journal.pone.0287643 (PMC10586641; doi:10.1371/journal.pone.0287643)
Supplement: Attachment — Submitted filename: Response to Reviewers.docx [file pone.0287643.s006.docx]

PLOS ONE

Editorial Office

<https://www.editorialmanager.com/pone>

**Subject: Point by point response to the manuscript PRCH-D-20-02039R3**

Dear, Alice Coles-Aldridge

I, the corresponding author, on behalf of the researchers would like to thank you for your extensive review to our manuscript," ***Factors associated with men involvement on attending antenatal care visits with their pregnant partners in Asmara, Eritrea: Community based survey*** "( **PONE-D-22-11568**). We appreciate your willing to consider our manuscript further once all the concerns have been addressed. Your concerns are addressed as per your recommendations and forwarding to you the responses point by point.

Yours Sincerely,

Ghirmay Ghebreigziabher Beraki

Corresponding author of the manuscript numbered PONE-D-22-11568

[gberaki83@gmail.com](mailto:gberaki83@gmail.com)

**Dear Reviewer,**

I, the corresponding author, on behalf of my co-authors would like to forward my appreciation for your constructive suggestions, corrections, comments and questions. Your concerns are addressed point by point. Thank you!

**Background**
The keywords do not match the mesh term

**Response:** Line 43: As per your recommendation, the keywords are modified in the new manuscript as follows. ‘Men involvement, ANC Partner attendant, affecting factors, Asmara’

Line 64-69: This paragraph is not consistent with the previous one and they are not related.
First, determine the important issue, is the prenatal visits the main issue or the participation of men in the visits? At the beginning of the statement of the problem, these items should be placed

**Response:** Line 45-51: Thank you**.** It is placed as per your recommendation in the first paragraph and the remaining paragraphs are thoroughly revised to address your concern.

Line 87: If there is no study in your country, but there are studies in other regions about participation barriers and factors related to the lack of participation of men, it is better to use these studies and enrich your problem statement.

**Response:** Line 81-97: Thank you**,** your comment well taken and addressed in last paragraph as follows:

‘Men participation in ANC is a form of health behaviour that is influenced by predisposing, supporting, and driving factors to reinforce behaviour change as suggested by Green et al. (1991) [17]. Studies indicated that factors reportedly associated with men involvement in ANC are significantly related to education, employment, income, access to media, number of children alive, ethnicity, religion, waiting time, staff information, and attitude [18-20]. Therefore, this study aims to assess the level of men involvement and analyse factors associated with men involvement in ANC in Asmara, Eritrea.’

**Methods**

Line 127: It had no other inclusion and exclusion criteria

**Response:** Line 122-125: It has inclusion and exclusion and it is restated as follows in the main manuscript. ‘Men of reproductive age (18-60 years) who had pregnant partners and/or had biological children aged up to 1 year at the time of data collection, had ANC attendant partner and willing to participate were included in the study. The 12 months’ grace period was employed to prevent recall bias from participants.’

Line 143: Quantitative content validity was not determined by CVI , CVR?

**Response:** Line 131-137: The quantitative content validity using content validity index (CVI) and content validity ratio (CVR) were not computed, instead, face content validity was assessed using a panel of experts of reproductive health from Asmara College of Medicine and Health Sciences as well as the Ministry of Health of the country. After evaluating the items, the acceptable items are preserved in the questionnaire, unacceptable items are removed, and modifiable items are revised and corrected by the panel of experts. This was done through cross checking. This meant that the researcher content validity measures contained all possible items that were used in measuring the concept. In this study, a valid measure of 0.5 was acceptable as in Spearman correlation coefficient.

Line 148: Did you have any questions about how to take care during pregnancy, the number and place of care, and other matters related to pregnancy, such as unwanted pregnancy, etc.?

**Response:** Line 150**-** 159: Since the care related, pregnancy related and other like questions are included at the items which are supposed to measure the knowledge of the participant, they were not included at the background information. Actually, instead of ‘background information’, we sensibly recognized to be called as ‘demographic background information’, and hence was amended accordingly at the revised manuscript.

Line 170: The number of questions related to male participation is very low. Did you have any questions about participating in visits, participating in housework, paying for attending the center, etc.?

**Response:** Questions about participating in visits and other necessary ones were included in the questionnaire (supplementary Table 3). To be specific, the variables that were included are listed below.

The last pregnancy of my partner was planned

My partner has visited ANC

I ever accompanied my partner to ANC

My partner has visited the ANC during her last pregnancy

I have accompanied my partner to ANC at least two times during her last pregnancy

Both me and my partner decide for her to seek ANC

Both me and partner arrange for her transportation

I escort my partner to ANC

I do discuss about pregnancy and child birth with other people

Line 179-180: This is an extra part. It is better to delete it

**Response:** As per the comment provided, the extra part is deleted in the new manuscript.

Line 184: The statement of the problem and the title are written in such a way that you are supposed to get the factors affecting the participation of men such as obstacles and improvement strategies, while in the following, knowledge, attitude and performance are given in the methods and the questionnaires are set accordingly. In this case, the title and statement of the problem and goals should be written in this direction.

**Response:** Line 180-185: As per the comment provided, necessary amendments are made at the new manuscript in such a way as to have consistency in the statement of the problem and goals of the study as follows:

‘Significance of association between potentially affecting factors of the respondents and male’s involvement was carried out using Chi square test of independence. Those variables with p<0.25 were further retained for multivariable logistic regression to predict the level of male’s involvement in ANC. This helped in identifying the net impact of each factor on the level of involvement of the respondents in ANC by controlling the effect of other factors in the model.’

Lin 190-194: Leave the reference of the definitions.

Response: Your comments are well taken and the operational definition section in revised manuscript is deleted.

**Results**

Line 188: Briefly explain the method of doing the work. How did men complete 100% of the questionnaires?

**Response:** Line 188-190: Thank you. ‘This study has a response rate of 94.21% at the initial visit. Participants not present initially were found at the second and third visits, by making convenient appointment, leading to successful inclusion of all selected partners.’ It is corrected accordingly.

**Discussion**
Line 297: This whole paragraph is extra. The discussion section should start with the purpose of the study and then your own findings.

**Response:** Comment accepted and the extra paragraph is deleted in the revised manuscript.

Line 303: The results of other studies should be mentioned the way of scoring the questionnaire has caused more people to have low knowledge and attitude

**Response**: Line 283-290: Thank you, this could be a reason to have low level of knowledge. We incorporated your suggestion in the revised manuscript and other studies are also cited to address your concern as follows.

‘This study considers the perspectives of male partner involvement in ANC and highlights the constraints and concern faced by them. Remarkable proportion of the respondents with low level of knowledge (43%) and attitude (42.5%) on ANC are the main concerns and constraints for level of men involvement (41.3%) based on the results of our study. Similarly knowledge level of participants was significantly associated as reported in similar studies in Indonesia (41.2 %) [17], Ethiopia (44.4%) [25] and a systemic review study [26]. Hence, to address the constraint perinatal health education interventions is needed to male partners in all reproductive age categories to improve their knowledge level on ANC.’

Line 318: One of the factors involved in the difference in the results of the studies is the questionnaires and how they are scored.

**Response:** Line 303-306: Thank you, well taken and incorporated in revised manuscript.

Line 329: The difference in attitude is more on which issue? You should mention these things in the comparison of findings.

**Response**: Line 312-319: You’re right, it is incorrectly stated. The paragraph is corrected in the revised manuscript as follows:

‘This cross sectional study identified that 57.5% of the respondents had positive attitude towards ANC which is less than the studies in Ethiopia (73.8%) [24] and Iran (65.3%) [28] but in line with that of Nigeria (56.5%) [32]. The main reason for having about average attitude level of the respondents in this study could be due to the influence of culture that considers maternal health as a women’s domain, lack of services targeting men and lack of privacy at the clinics. Therefore, there is a need to bring a change in men’s attitude to have a greater understanding in maternal health care outcome because men are the main decision-makers of health care in families in Eritrea.’

Line 363: According to the way of conducting the study, this goal is not correct and the goal and title of the study should be changed.

**Response:** Line 349-351: The study used both bivariate and multivariate analysis which identified factors associated with the male involvement in attending ANC with their partners. Sorry for not clearly stated the strength of study. It is corrected to avoid the confusion with the goal and title of the study as follows: ‘Having adequate sample size for the estimation of the male’s involvement in ANC is the major strength of the study. However, as the study is a cross-sectional, there is a difficulty to draw causal inferences.’

With regards,

Ghirmay

Corresponding Author
