# Supplementary material for: Factors associated with men’s involvement in antenatal care visits in Asmara, Eritrea: Community-based survey
Source: PLoS One. 2023 Oct 19;18(10):e0287643. doi: 10.1371/journal.pone.0287643 (PMC10586641; doi:10.1371/journal.pone.0287643)
Supplement: Attachment — Submitted filename: Review_PONE-D-22-11568.docx [file pone.0287643.s007.docx]

**Paper Title:** Factors associated with men’s involvement in attending antenatal care visits with their pregnant partners in Asmara, Eritrea: Community based survey**.**

Reviewer suggested title: Factors associated with men’s involvement in antenatal care visits in Asmara, Eritrea: Community-based survey.

| **No.** | **Reviewer’s Comment** |
| --- | --- |
| **1.** | **THEORETICAL FOUNDATION/GENERAL INFORMATION** |
| i) | **Clarity of problems being investigated** |
|  | The study investigated male partner involvement in antenatal care visits in Asmara, Eritrea. The study provided a justification why partner involvement in maternal health services helps health promotion strategies to improve maternal and child health. However,   i. The study failed to provide statistics on partner involvement in ANC visits in Eritrea. What is the current statistic of male involvement in Eritrea? |
| **2.** | **THE DESIGN AND METHODOLOGY USED** |
| i. | The study used a cross-sectional design. Results are presented in univariate, bivariate and multivariate analysis, which is very appropriate.  However, the study lacks clarity on the unit of analysis. Did the study sample male respondents for interviews or households? If there were more than one household member whose partners were pregnant, were they qualified for the interview? How were the households of the males selected? |
| ii. | There is a methodological discordance as presented by the authors. Which method did the study actually use? Is it purposive or systematic? More clarity needs to be provided on which stage each method was used. |
| iii. | The study failed to provide justification for the site selection. What are the main parameters that were considered in the selection of the study site? |
| iv. | The study categorization of knowledge of ANC is problematic and must be reviewed. The study measurement of knowledge as poor and good must be reworked. Additionally, the study claimed that “Respondents with a total score below the mean were considered to have a poor level of knowledge, and those with a total score above the mean were considered to have a good level of knowledge on ANC”. What about the respondents who had an average or mean score? I am thinking that all the men or respondents in Asmara, Eritrea could not be lumped as having poor and good knowledge of ANC, some could have moderate knowledge. |
| v. | Lines 157 to 159 must be reworked. |
| vi. | Lines 167 to 169 on the measurement of attitudes toward ANC must be reworked. I do not see where to place respondents who had exactly the mean or average score. I am thinking that all the men or respondents in Asmara, Eritrea could not be lumped as having negative and positive attitudes toward ANC, some could have neutral attitudes as well. |
| vii. | Lines 172 to 175 should be revised. The total marks score for a respondent if he had been involved in all the ANC-related statements should be more than 7 marks. Again, the measurement of involvement or ANC practices should be revised. The two-level categorization is problematic and does not provide clarity. How about men who have a fair level of involvement? |
| viii. | The authors might like to read Bloom BS. (1956) Taxonomy education. New York: David McKay. This could help to firm up the assessment of the overall respondents’ KAP (knowledge, attitude and practices) of ANC level based on the Bloom’s cut-off. |
| **3.** | **ANALYSIS AND PRESENTATION** |
| i. | The Level of education variable must be revised to meet the five standard levels of education in Eritrea. |
| ii. | The total number of respondents is 605. However, in line 203, two variables Respondent’s current occupation and Partner’s occupation have less than the total sum. Where are the rest of the respondents? |
| iii. | There is no clarity in the multivariate analysis. The authors should consider presenting a separate table for the multivariate analysis. It appears the authors have lumped bivariate and multivariate analysis. Table 4 is a bit confusing to the reader. |
| iv. | The chi-square (x^2^) statistic should be added in the bivariate result table in Table 3. This should be revised. |
| **4.** | **MAJOR FINDINGS FROM THE RESEARCH** |
| i. | Religion, educational level and level of ANC knowledge significantly influence partner involvement in ANC visits. |
| **5.** | **Conclusion Based on Data** |
| i. | The conclusion is problematic and self-contradictory. In line 244 and 245, the study presented in Table 2, that 57.0%, 57.5%, and 58.7% of the respondents scored more than or equal to the mean level of knowledge, attitude, and involvement in ANC, respectively. However, in line 368 and 369, the authors wrote that; “the study showed that close to sixty percent of the respondents had a good level of knowledge, attitude, and involvement in ANC.” In another breath, the study concluded in line 39 that, “male partner involvement in ANC was considerably low”. This need to be revised. |
| **6.** | **GENERAL COMMENTS** |
|  | On the whole, the paper is a good piece. However, the following must be noted and revised: |
|  | (a) The title of the study should be refined as suggested earlier. |
|  | (b) The authors need to firm up the justification for selecting the study site. |
|  | (c) More explanation needs to be provided on the multistage sampling to include how the community level was selected from the sub-administration |
