# Supplementary material for: Factors associated with men’s involvement in antenatal care visits in Asmara, Eritrea: Community-based survey
Source: PLoS One. 2023 Oct 19;18(10):e0287643. doi: 10.1371/journal.pone.0287643 (PMC10586641; doi:10.1371/journal.pone.0287643)
Supplement: Attachment — Submitted filename: Response to Reviewers .docx [file pone.0287643.s008.docx]

Martin Wiredu Agyekum, PhD

Guest Editor

PLOS ONE

<https://www.editorialmanager.com/pone>

**Subject: Submission point by point response to the manuscript PONE-D-22-11568R1**

Dear, Dr. Martin

Thank you for the extensive review and opportunity to submit a revised version of our manuscript. We appreciate the editor and reviewers’ dedication for the extensive and detailed assessment of the manuscript and thoughtful comments. In responding to the comments, we have made a point-by-point revision of the manuscript to reflect most of the suggestions given and rebuttal for the rejected ones, if any. We have highlighted the changes within the manuscript in track changes. We hope this revised version of the manuscript fully meets PLOS ONE’s publication criteria.

We sincerely thank you for considering this manuscript and look forward to your positive responses.

Best regards,

Ghirmay Ghebreigziabher Beraki, MSc

Assistant Professor, Orotta College of Medicine and Health Sciences

Corresponding author of the manuscript numbered [PONE-D-22-11568R1]

[gberaki83@gmail.com](mailto:gberaki83@gmail.com)

## **Response to Editor‘s comment**

**Response**: Line: 1-2: Thank you, comment is well noted. The title is revised accordingly. ‘Factors associated with men involvement in antenatal care visits in Asmara, Eritrea: Community based survey’.

## **Response to Reviewer # 2**

**Dear David,**

On behalf of my co-authors, I would like to forward my appreciation for your constructive suggestions, corrections, comments and questions. I am glad to present the point by point responses stated blow to reflect most of the concerns raised to look forward to your positive feedback. Thank you.

Sincerely,

Ghirmay

1. **Paper Title:** Factors associated with men’s involvement in attending antenatal care visits with their pregnant partners in Asmara, Eritrea: Community based survey**.**

Reviewer suggested title: Factors associated with men’s involvement in antenatal care visits in Asmara, Eritrea: Community-based survey.

- **Response**: Line: 1-2: Thank you; it is corrected as per your suggestion.

“Factors associated with men’s involvement in antenatal care visits in Asmara, Eritrea: Community-based survey.”

| **No.** | **Reviewer’s Comment** |
| --- | --- |
| **1.** | **THEORETICAL FOUNDATION/GENERAL INFORMATION** |
| i. | **Clarity of problems being investigated** |
|  | The study investigated male partner involvement in antenatal care visits in Asmara, Eritrea. The study provided a justification why partner involvement in maternal health services helps health promotion strategies to improve maternal and child health. However,   i. The study failed to provide statistics on partner involvement in ANC visits in Eritrea. What is the current statistic of male involvement in Eritrea?  - **Response**: Line: 35 & Line 256-258: Thank you. Your question is addressed. This study provided male partner level of involvement in ANC visits was 58.7% in Asmara-Eritrea. |
| **2.** | **THE DESIGN AND METHODOLOGY USED** |
| ii. | The study used a cross-sectional design. Results are presented in univariate, bivariate and multivariate analysis, which is very appropriate.  However, the study lacks clarity on the unit of analysis. Did the study sample male respondents for interviews or households? If there were more than one household member whose partners were pregnant, were they qualified for the interview? How were the households of the males selected?   - **Response**: Household with pregnant woman/women with child/children under one year of age was selected for the study. Male partner residing in the selected households were, however, the unit of analysis of the study. If there were more than one household member whose partners were pregnant, they were all qualified for the interview. Unfortunately, there was no selected household with more than one pregnant woman in the study area during the study period. |
| iii. | There is a methodological discordance as presented by the authors. Which method did the study actually use? Is it purposive or systematic? More clarity needs to be provided on which stage each method was used.   - **Response**: Line 119-131: Thank you, comment is well noted. You are right; previously, it seemed that the sampling method used to select the cluster from each administration area looks as if purposive. However, now it is written clearly.   - At the first stage, selection of administration area, simple random sampling was used.   - At the second stage, systematic random sampling was used to randomly select households from the list of households who had male partner of pregnant women or infants from the list of the selected administration area. |
| iv. | The study failed to provide justification for the site selection. What are the main parameters that were considered in the selection of the study site?   - **Response**: Line: 96-98: You raised very interesting point, thank you. The study site is selected by virtue of it being a capital city of the country in which male involvement is currently highly advertised, as a result of which the awareness raisings and their practical applicability need to be gauged numerically. |
| v. | The study categorization of knowledge of ANC is problematic and must be reviewed. The study measurement of knowledge as poor and good must be reworked. Additionally, the study claimed that “Respondents with a total score below the mean were considered to have a poor level of knowledge, and those with a total score above the mean were considered to have a good level of knowledge on ANC”. What about the respondents who had an average or mean score? I am thinking that all the men or respondents in Asmara, Eritrea could not be lumped as having poor and good knowledge of ANC, some could have moderate knowledge.   - **Response**: Line: 163-165: Thank you for your correction. Even though not specifically stated in the method section of the manuscript, it was stated in Table 2 of the analysis results, we have classified those respondents with total knowledge score equal to the mean as having good level of knowledge on ANC.   Actually, it is true that the categorization of the individuals’ knowledge is very controversial all the time. Sometimes, it might not even be advisable to divide into two as ‘good’ or ‘poor’; other times, it might not be preferable to classify it as ‘good’, ‘moderate’, and ‘poor’. Sometimes, it might not be advisable to use the mean or median, and other times it might not be advisable to use some fixed standardized references for the study setting might differ.  However, the ‘objective’ of the study is usually used as a rule of thumb to make the classification (either to two or three), which in this case the researchers were mainly focusing to determine factors associated with good knowledge among the men as compared to those individuals having poor knowledge. If the categories are made as three, the factors might not clearly be portrayed to the audience for the analysis will be made using multinomial logistic regression. And most of the time, the results of the multinomial logistic regression are not friendly to the readers. |
| vi. | Lines 157 to 159 must be reworked.   - **Response**: Line: 163-165: Thank you. It is corrected accordingly. |
| vii. | Lines 167 to 169 on the measurement of attitudes toward ANC must be reworked. I do not see where to place respondents who had exactly the mean or average score. I am thinking that all the men or respondents in Asmara, Eritrea could not be lumped as having negative and positive attitudes toward ANC, some could have neutral attitudes as well.   - **Response**: Line: 173-175: Thank you for your correction. Even though not specifically stated in the method section of the manuscript, it was stated in Table 2 of the analysis results, we have classified those respondents with total attitude score equal to the mean as having positive attitude on ANC.   The attitude response classification in two (‘positive and negative’ rather than to three (positive, neutral and negative’) is due to similar reason given as in the above ‘Response iv’. |
| viii. | Lines 172 to 175 should be revised. The total marks score for a respondent if he had been involved in all the ANC-related statements should be more than 7 marks. Again, the measurement of involvement or ANC practices should be revised. The two-level categorization is problematic and does not provide clarity. How about men who have a fair level of involvement?   - **Response**: Line: 181-183: Thank you for your correction. Even though not specifically stated in the method section of the manuscript, it was stated in Table 2 of the analysis results.   The involvement response classification in two (‘good and poor’) rather than to three (‘good, fair and poor’) is due to similar reason given as in the above ‘Response iv’. |
| ix. | The authors might like to read Bloom BS. (1956) Taxonomy education. New York: David McKay. This could help to firm up the assessment of the overall respondents’ KAP (knowledge, attitude and practices) of ANC level based on the Bloom’s cut-off.   - **Response**: We appreciate your deep search to enhance our manuscript quality. Thank you for the reference material too. However, due to the reasons given above in ‘Response iv’ the survey used the two cut-off option rather than three (Bloom’s cut-off) to best fit for the objectives and analysis of the study. |
| **3.** | **ANALYSIS AND PRESENTATION** |
| i. | The Level of education variable must be revised to meet the five standard levels of education in Eritrea.   - **Response**: Yes you are right and as it is stated in Table 1, the five levels of education categories were stated separately. However, in the bivariate and multivariate analysis tables, the first three levels were lumped together into one category “below secondary”. This was mainly to have adequate number of cases required by the bivariate (chi-square) and multivariate (logistic regression) analysis and get reliable results. |
| ii. | The total number of respondents is 605. However, in line 203, two variables Respondent’s current occupation and Partner’s occupation have less than the total sum. Where are the rest of the respondents?   - **Response**: Line: 212-214: You are right. The unemployed respondents and partners were excluded from the Table 1. But now included and corrected accordingly. |
| iii. | There is no clarity in the multivariate analysis. The authors should consider presenting a separate table for the multivariate analysis. It appears the authors have lumped bivariate and multivariate analysis. Table 4 is a bit confusing to the reader.   - **Response**: Line: 297-302: Accepted, Table 4 was split into Table 4a and 4b for bivariate and multivariate analysis results, respectively. |
| iv. | The chi-square (x^2^) statistic should be added in the bivariate result table in Table 3. This should be revised.   - **Response**: Line: 272: Well accepted and revised according to your suggestion. |
| **4.** | **MAJOR FINDINGS FROM THE RESEARCH** |
| i. | Religion, educational level and level of ANC knowledge significantly influence partner involvement in ANC visits.   - **Response**: Thanks and your comment is well noted. |
| **5.** | **Conclusion Based on Data** |
| i. | The conclusion is problematic and self-contradictory. In line 244 and 245, the study presented in Table 2, that 57.0%, 57.5%, and 58.7% of the respondents scored more than or equal to the mean level of knowledge, attitude, and involvement in ANC, respectively. However, in line 368 and 369, the authors wrote that; “the study showed that close to sixty percent of the respondents had a good level of knowledge, attitude, and involvement in ANC.” In another breath, the study concluded in line 39 that, “male partner involvement in ANC was considerably low”. This need to be revised.   - **Response**: Line: 378-387: Thanks and the conclusion is revised thoroughly.   Male partner level of involvement in ANC visits in Asmara-Eritrea was generally good. Nonetheless, most of the respondents believed that pregnancy is a female domain. This belief can pose a challenge to the involvement of males in ANC. Therefore, there is a need to create an outlook that favors an equal share of responsibilities during pregnancy.  The associated factors in hindering male involvement in ANC visits in this study were level of education, religious affiliation and level of knowledge. Therefore, special emphasis should be given to male partners who read and write illiterates, elementary and junior level of education, higher level of education, and Muslims. Hence, educational and religious institutions can be used as a platform for health promotion strategy to enhance male partner involvement in ANC visits to improve maternal and child health outcomes. |
| **6.** | **GENERAL COMMENTS** |
|  | On the whole, the paper is a good piece. However, the following must be noted and revised: |
|  | (a) The title of the study should be refined as suggested earlier.   **Response**: Thank you, it is correct as per your suggestion. |
|  | (b) The authors need to firm up the justification for selecting the study site.   **Response**: Well noted, the selection criteria are thoroughly revised. |
|  | (c) More explanation needs to be provided on the multistage sampling to include how the community level was selected from the sub-administration   **Response**: Accepted, sampling methods are thoroughly revised and corrected. |

## **Response to Reviewer #3:**

Dear authors, thank you for writing this manuscript entitled ‘’ Factors associated with men’s involvement in attending antenatal care visits with their pregnant partners in Asmara, Eritrea: Community based survey’’

**Dear Reviewer #3,**

We the authors, appreciate your comprehensive assessment of the manuscript, productive suggestions and encouragement. We are gladly to accept and include your suggestions and comments in the revised manuscript.

With regards,

Ghirmay

**Title**

1. I found that this manuscript is well written. However, I have a few comments listed below. Title can be modified: into ‘prevalence of men’s involvement and its associated factor……Think over it.

- **Response**: Line: 1-2: Thank you for your suggestion. Suggestion was also given by the other review and editor regarding the title. Based on your suggestions the title is revised as follows:

Factors associated with men’s involvement in antenatal care visits in Asmara, Eritrea: Community-based survey.

2. This study will be interesting if it was conducted in a hospital, why did you chose community based study, how do you know the true prevalence of male involvement at the community level?

- **Response**: Yes, it could be possible to do the study in health facilities. However, most of the time pregnant women come to ANC visits unaccompanied by their partners according to our observation. Hence, there will be limited chance of getting male partners for interview during the ANC visits.

Regarding the true prevalence of male involvement at the community level in Asmara, because the sample was randomly selected and representative of all male partners in Asmara city, the estimate we get is reliable and represents the true prevalence with reasonable margin of error of 2% and 95% Confidence Interval of (54.7%, 62.5%).

**Abstract**

1. How many objectives do you have? Line 24 25 showed that only one objective factors associated with male involvement what about the prevalence of male involvement?

- **Response**: Line: 23-24: Thank you for identifying the error and the manuscript is revised to integrate your correction. The objects are mainly two. The first one is estimating the level of male partners’ involvement in ANC visits and the second objective is to identify the factors associated with the male involvement.

2. What is the prevalence of Male involvement?

- **Response**: Thank you for reminder us the point.

Line 35: The study estimated the prevalence of 58.7% men involvement in ANC visits with margin of error and 95% confidence interval indicated above.

3. The authors were listed only p value, it will be better if you add the adjusted odds ratio, CI etc.

- **Response**: Accepted. It is revised accordingly in the abstract section as well in the results section in Table 4b.

Abstract, Line: 35-37: Religion (p=0.006, AOR=1.91, 95% CI 1.20-3.03), level of education (p=0.027, AOR=1.96, 95% CI 1.08-3.57), and level of knowledge (p<0.001, AOR=3.80, 95% CI 2.46-5.87) were significant associated factors of male involvement in ANC.

4. The conclusion section needs revision, what is below and above secondary levels of education?

- **Response**: Thank you, comment is well noted and revised as follows:

Line: 41-43: Male partner level of involvement in ANC visits in Asmara was generally good. The associated factors in hindering male involvement in ANC visits were level of education, religious affiliation and level of knowledge. Hence, educational and religious institutions will be a good platform for health promotion strategy to enhance male partner involvement in ANC visits to improve maternal and child health outcomes.

5. The authors used predictors, factors associated with, factors affecting…be consistent use one words

- **Response**: Well taken. It is correct as ‘factors associated with’ throughout the text.

**Background**

1. This section needs extensive revision, for instance line 53 to 57, start from WHO, then Eritrea!

- **Response**: Comment well taken, the background is revised thoroughly.

Line: 55-62: The World Health Organization (WHO) estimated that globally 295,000 women die annually as a result of complications of pregnancy and childbirth. Sub-Saharan Africa and Southern Asia accounted for approximately 86% of the estimated global maternal deaths, with sub-Saharan Africa alone accounting for roughly 66% [5]. Eritrea's estimated maternal mortality ratio is still unacceptably high (480/100,000 live births) [5]. Therefore, to reduce the high mortality rates in developing countries like Eritrea, involvement of men is crucial to achieving Sustainable Development Goals, with a target of 70 per 100,000 live births in 2030 [6].

**Results**

1. It is better to say socio demographic characteristics rather than Demographic background information.

- **Response**: Comment well taken, the manuscript was revised accordingly.

2. How many questions used to assess knowledge and attitude? And it will be wonderful if you included each variable used to assess knowledge and attitude using table.

- **Response**: Line: The knowledge and attitude questions are attached as supplementary information to reduce the number of tables in the manuscript.

3. Use only one table for bivariate and multivariate analysis. And also I did not see the crude odds ratio?

- **Response**: Line: 297-302: Your comment is well taken. Separate tables are presented for bivariate and multivariate analysis results. We included the adjusted odds ratio in the multivariate results (Table 4b) and the crude odds ratio and their 95% confidence interval in Table 4a (bivariate analysis results). The crude odds ratio values are obtained by running bivariate logistic regression analysis. In the bivariate analysis we used chi-square procedure which provides chi-square value and p-value to help us decide which factors to include in the multivariate analysis.

4. In table four the author stated others, what are others you have to write at the end of the table.

- **Response**: Line: 299: Thank you for asking the clarification. Others category under marital status include: single and cohabiting and it is stated as per your recommendation.

**Discussion**

It is well written

- **Response**: Thank you!

Strength and limitation of the study needs revisions

- **Response**: Your suggestion is well taken and the strength and limitation of the study is revised to address your concern as follows:

Line:370-376: The survey was capable to estimate the prevalence of men involvement in ANC visits at community level in Asmara-Eritrea, with adequate sample size, which can be useful for maternal and child health planners. However, the study cannot determine the causal relationship because of lack of chronological order of the data collected for the exposure and outcome variables. The participants are also prone to response bias in the sense that the replies are recorded as per the self-response of the individuals. It is known that the self-responses are prone to cultural, moral and social acceptable activities and ideas.
